# Study on the Application of Super-Resolution Ultrasound for Cerebral Vessel Imaging in Rhesus Monkeys

**DOI:** 10.3389/fneur.2021.720320

**Published:** 2021-11-17

**Authors:** Li Yan, Chen Bai, Yu Zheng, Xiaodong Zhou, Mingxi Wan, Yujin Zong, Shanshan Chen, Yin Zhou

**Affiliations:** ^1^Institute of Medical Research, Northwestern Polytechnical University, Xi'an, China; ^2^State Key Laboratory of Transient Optics and Photonics, Xi'an Institute of Optics and Precision Mechanics, Chinese Academy of Sciences, Xi'an, China; ^3^Department of Ultrasonography, Xi'an Central Hospital, The Third Affiliated Hospital of Jiaotong University, Xi'an, China; ^4^Ultrasound Diagnosis & Treatment Center, Xi'an International Medical Center, Xi'an, China; ^5^Key Laboratory of Biomedical Information Engineering of Ministry of Education, Department of Biomedical Engineering, Xi'an Jiaotong University, Xi'an, China

**Keywords:** super-resolution imaging, ultrasound, cerebral vessel, microbubbles, monkey

## Abstract

**Background:** Ultrasound is ideal for displaying intracranial great vessels but not intracranial microvessels and terminal vessels. Even with contrast agents, the imaging effect is still unsatisfactory. In recent years, significant theoretical advances have been achieved in super-resolution imaging. The latest commonly used ultrafast plane-wave ultrasound Doppler imaging of the brain and microbubble-based super-resolution ultrasound imaging have been applied to the imaging of cerebral microvessels and blood flow in small animals such as mice but have not been applied to *in vivo* imaging of the cerebral microvessels in monkeys and larger animals. In China, preliminary research results have been obtained using super-resolution imaging in certain fields but rarely in fundamental and clinical experiments on large animals. In recent years, we have conducted a joint study with the Xi'an Jiaotong University to explore the application and performance of this new technique in the diagnosis of cerebrovascular diseases in large animals.

**Objective:** To explore the characteristics and advantages of microbubble-based super-resolution ultrasound imaging of intracranial vessels in rhesus monkeys compared with conventional transcranial ultrasound.

**Methods:** First, the effectiveness and feasibility of the super-resolution imaging technique were verified by modular simulation experiments. Then, the imaging parameters were adjusted based on *in vitro* experiments. Finally, two rhesus monkeys were used for *in vivo* experiments of intracranial microvessel imaging.

**Results:** Compared with conventional plane-wave imaging, super-resolution imaging could measure the inner diameters of cerebral microvessels at a resolution of 1 mm or even 0.7 mm and extract blood flow information. In addition, it has a better signal-to-noise ratio (5.625 dB higher) and higher resolution (~30-fold higher). The results of the experiments with rhesus monkeys showed that microbubble-based super-resolution ultrasound imaging can achieve an optimal resolution at the micron level and an imaging depth >35 mm.

**Conclusion:** Super-resolution imaging can realize the monitoring imaging of high-resolution and fast calculation of microbubbles in the process of tissue damage, providing an important experimental basis for the clinical application of non-invasive transcranial ultrasound.

## Introduction

Among all kinds of medical imaging modes, ultrasound imaging stands out for its real-time dynamics, high safety, non-invasiveness, convenience, and low cost (1). However, conventional B-mode ultrasound imaging or contrast-enhanced ultrasound (CEUS) imaging in clinical practice is restricted by fundamental diffraction limits, and the resolution therefore cannot reach the submillimeter scale. Thus, conventional B-mode ultrasound imaging and CEUS imaging cannot accurately and clearly display microvascular structures (such as the microvasculature) of subwavelength size. At present, super-resolution ultrasound imaging successfully overcomes the half-wavelength resolution limit by obtaining microbubble (MB) positions from radiofrequency data, positioning, and tracking the continuous movement trajectory of MBs using an algorithm, and reconstructing the blood flow information in microvessels. For example, ultrasound localization microscopy (2) forms a final image by localizing spatially separated individual MBs using multiple frames. Viessmann et al. (3) demonstrated that a clinical CEUS system with a central frequency of 2 MHz can realize the imaging of two parallel tubes with an inner diameter of 200 μm.

In recent years, significant theoretical advances have been achieved in super-resolution imaging. At present, ultrafast plane-wave ultrasound Doppler imaging of the brain (4) and MB-based super-resolution ultrasound imaging (5) have been applied to the imaging of the interior of microfluidic channels (6) and synchrotron radiation imaging of microvessels and tissue models of *in vitro* human skulls (7) and *in vivo* mouse and rat head models (5, 8–11), but have not been applied to *in vivo* imaging of the cerebral microvessels in monkeys and larger animals. In China, although preliminary research results have been obtained using super-resolution imaging in certain fields, the limited conditions for fundamental and clinical experiments on large animals complicate the execution of super-resolution ultrasound imaging experiments on large animals *in vivo*. Therefore, we conducted a joint study with Xi'an Jiaotong University to explore the application and performance of this new technique in cerebrovascular imaging and disease diagnosis in large animals.

## Materials and Methods

### Instruments

The ultrasound machine used in the study was a VINNO G50 (VINNO Technology Co., Ltd., China) and a 3M phased array probe was employed for conventional imaging. The super-resolution ultrasound research system was Vantage 256 from Verasonics (Verasonics, Inc. Washington, United States), and a self-developed 128-channel low-frequency linear array probe (12, 13) was used for the super-resolution ultrasound imaging.

### Experimental Animals

With the approval of the Institutional Animal Care and Use Committee of the Air Force Medical University, the study subjects were two male rhesus monkeys (4.7 kg, 5.2 years; 8.6 kg, 7.5 years), which were purchased from Guangxi Fangchenggang Spring Biological Technology Development Co., Ltd. (license No. SCXK Gui 2013-0004) after the approval.

### Imaging and Experimental Methods

#### High-Sensitivity Super-Resolution Ultrasound Imaging Method for Cerebral Microvessels (R&D by the Xi'an Jiaotong University)

Low-frequency chirp waves can be effective and useful to increase the time-bandwidth product of a signal by changing the time and frequency bandwidths of the ultrasonic transmitting signal. Its purpose is to enhance the energy of the signal such that it can penetrate the skull to obtain intracranial information for imaging. Multiangle compound imaging is a common method used to improve image quality and contrast in plane-wave imaging.

**Figure 1:**
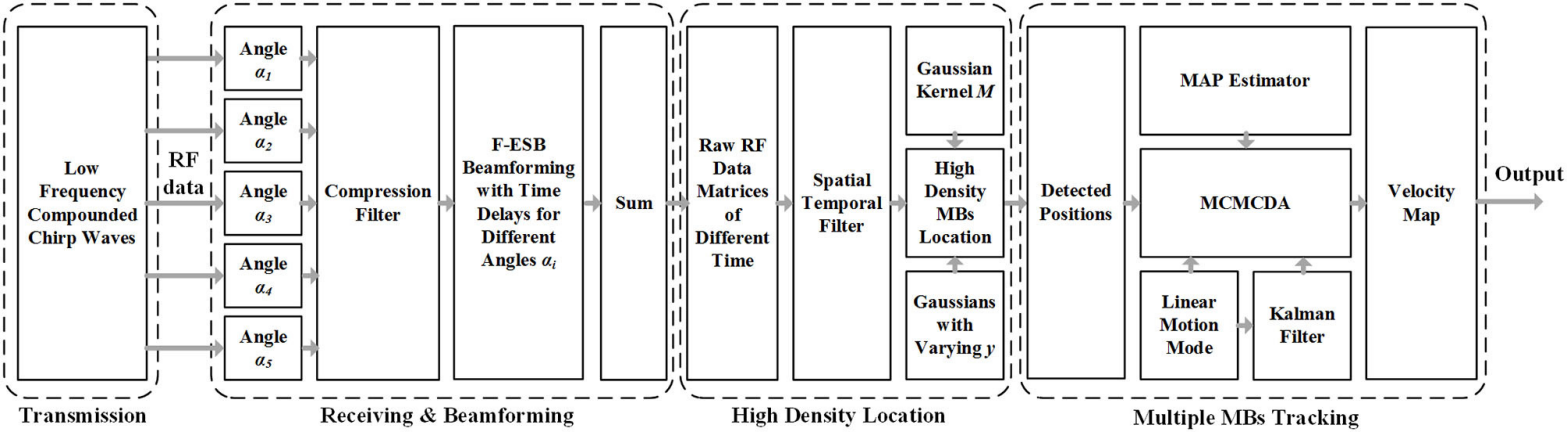
Schematic view of transcranial ultrasound vascular imaging (TUVl) based on low frequency compounded chirp waves and the Markov chain Monte Carlo data association (MCMCDA) tracking algorithm. The compounded chirp waves enhance the sensitivity of the microbubble (MB) detection process while the Fast eigenspace-based beamforming (F-ESB) improves the fundamental imaging quality with low computational complexity. Based on the results of the spatial-temporal filtering, high-density MBs can be located. The map of the final vessels with blood flow velocity is achieved by tracking the detected MBs based on the MCMCDA algorithm.

A research group at the Xi'an Jiaotong University (14) proposed a super-resolution imaging method for cerebral microvessel combining low-frequency ultrasound, chirp waves, and multiangle compound imaging. The method reconciles the conflict between imaging depth, detection sensitivity, and imaging resolution caused by skull shielding by using compound chirp waves (CCWs) as the signal emission mode. On this basis, the modified Markov chain Monte Carlo data association (MCMCDA) algorithm was used to track multiple high-concentration MBs (contrast agents) to realize the super-resolution imaging of the micron-level cerebral microvascular structure and blood flow information. The method based on a 5-angle CCW (CCW-5) with low frequency and MCMCDA algorithm was applied to the channel domain of the radiofrequency (RF) data. Figure 1 provides the schematic representation of the entire data processing pipeline of our proposed method. The pipeline contains the following components: (a) ultrafast compounded chirp waves with a low frequency; (b) detection and tracking of multiple MBs.

a) *Ultrafast CCW With Low Frequency*

Chirp excitation uses a longer short pulse waveform along with pulse compression of the backscattered signals to increase the penetration depth and the signal-to-noise ratio (SNR) of MBs detection while retaining the axial resolution (15). In addition, the compounded imaging technique mode for ultrasound imaging can provide high-resolution image quality with a high temporal resolution (16). Therefore, in our work, we combined the chirp waves with compounded imaging, i.e., CCW, to improve the sensitivity of the transcranial detection, increasing the imaging contrast.

b) *Detection and Tracking of Multiple MBs*

The process consists of three steps: (1) detection of the positions of the MBs; (2) setting of the motion model; (3) tracking of multiple MBs. The velocities of the estimated MB in the different trajectories are associated with their positions in the state vector. Then, these are normalized and mapped onto the vessels to provide additional information to the observer. Compared with the speckle tracking method, the determination of the flow velocities in super-resolution is advantageous as it allows for the display of the blood flow in the microvessels.

#### Evolution by Simulations and Phantom Experiments

**Figure 2:**
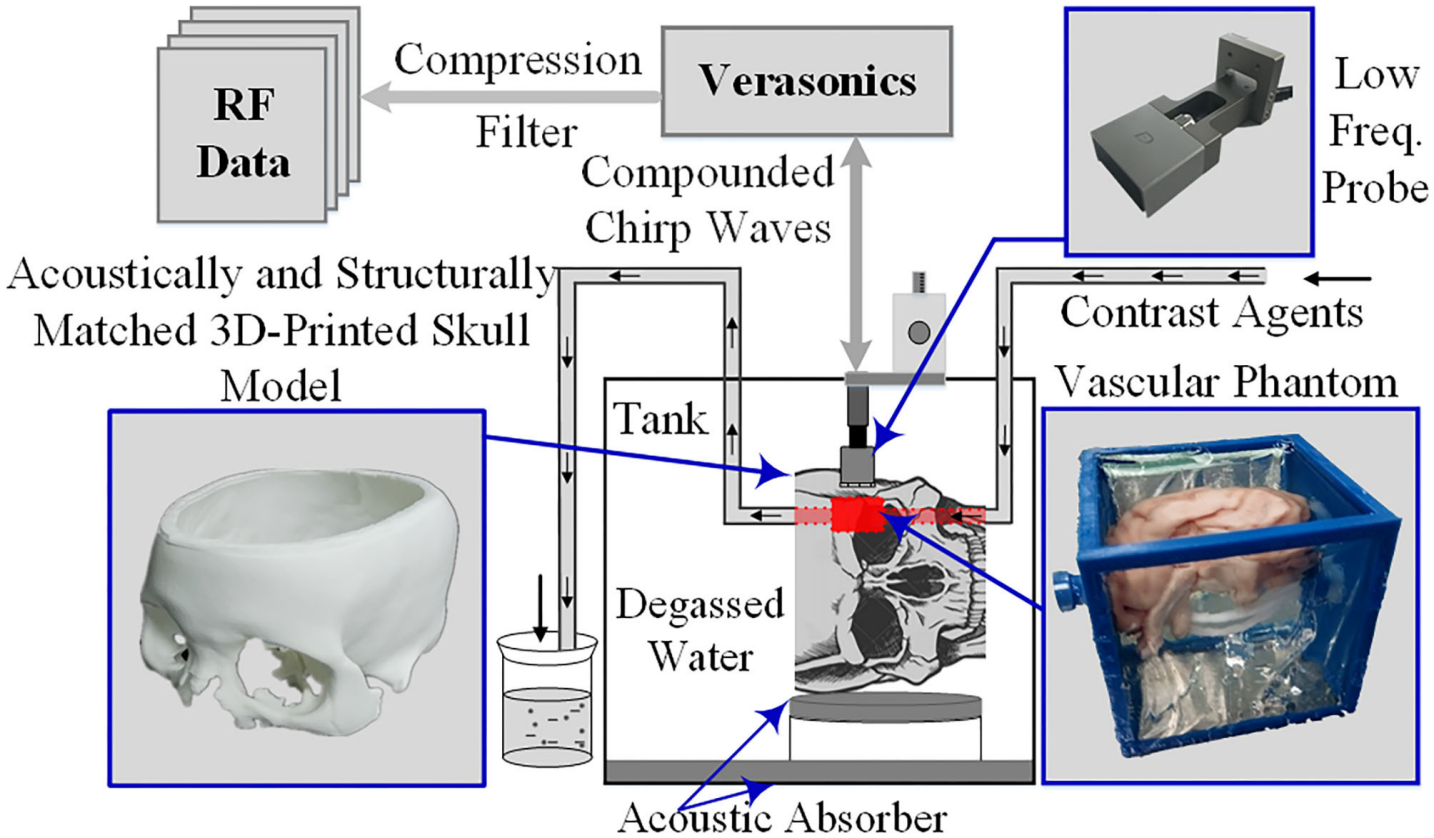
Phantom imaging experimental framework.

The super-resolution imaging method was further verified using phantom experiments following internationally accepted standards. Acoustically and structurally matched three-dimensional printed skull models as well as vascular phantoms were used in the experiments to simulate the real skull and intracranial tissue environment (17). The phantom experiments were performed in a rectangular polycarbonate tank filled with degassed water. The inner wall and the bottom of the tank were also covered with sound-absorbing material to minimize the acoustic reflection caused by the tank. As shown in Figure 2, the 128-channel linear-array transducer is matched and connected to a digital programmable ultrasound imaging system (Vantage 256, Verasonics Inc., Kirkland, Washington, United States), and a three-dimensional fixed-axis platform is placed on top of the tank to fix the imaging probe and precisely control its position. The contrast agent MBs (SonoVue, Bracco Spa, Milan, Italy) were diluted by ~1,000 times, injected into the container filled with degassed water, and circulated at a rate of ~30 mm/s in a circulatory system composed of a rubber tube and a curved and bifurcated vessel model, powered by a constant-flow pump. Subsequently, the five-angle compound imaging sequence (CCW-5, −6°, −3°, 0°, 3°, and 6°, frame rate 1,500 Hz) was obtained in the experimental system for linear frequency-modulated signals (sweep range 1.3–2.7 MHz, time-bandwidth 6 μs).

#### Super-Resolution Imaging of Cerebral Microvessels in Rhesus Monkeys *in vivo*

**Figure 3:**
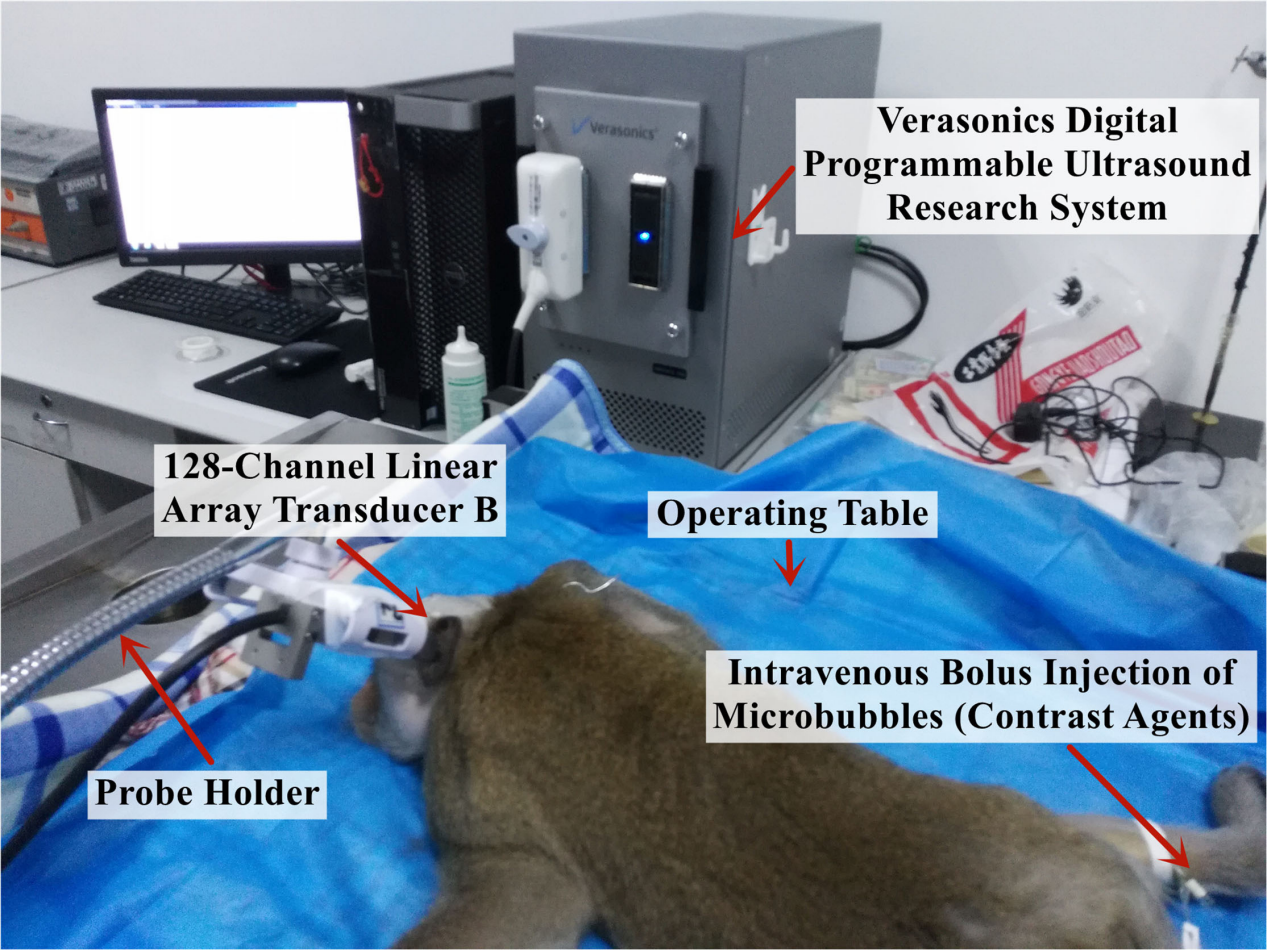
Experimental platform for the *in vivo* imaging of the brain microvessels in rhesus monkeys.

The rhesus monkeys used in the study were fasted for 12 h and water-deprived for 8 h before surgery. Intraperitoneal anesthesia was performed using a 3% sodium pentobarbital solution at 1 mL/kg, and half of the initial drug dose was injected every 2 h to maintain the anesthesia during the experiment. After each monkey was thoroughly anesthetized, it was fixed on the operating table in a prone position and heated using an electric blanket to ensure a stable body temperature at ~38°C throughout the experiment (Figure 3).

First, to perform the perfusion imaging of the monkey brain microvessels, the contrast agent MBs (SonoVue, Bracco Spa, Milan, Italy) were diluted with normal saline to a concentration equivalent to ~500,000 MBs/ml per injection, and the injection dose was judged by clinical experience at a ratio equivalent to the injection dose used clinically in adults. Subsequently, the ultrasound imaging probe scanned the brain microvessels of the monkeys using a linear frequency-modulated multiangle compound emission mode (1.3–2.7 MHz, time-bandwidth 6 μs), and the computer experimental platform implemented the MCMCDA algorithm to obtain high-sensitivity and super-resolution imaging results. Contrast agent injection and cerebrovascular imaging data acquisition were performed every 15 min during the experiment to ensure the complete metabolism of the previously injected MBs.

The 2M low-frequency linear-array probes (128 channels) developed by the Xi'an Jiaotong University were used and fixed with a steel frame and a universal clamp. After selecting the appropriate position through image comparisons, the probes were each placed near the fontanelle of the rhesus monkeys and on the temporal bone region. The ultrasound imaging data were collected using the digitized programmable ultrasound imaging system described previously, with a mechanical index maintained at 0.6 to ensure that the signal penetrated the skull without rupturing the MBs and damaging the intracranial tissue, a frame rate of 300 Hz, and an acquisition time of 4 s for each imaging session, i.e., a total of 1,200 frames of radiofrequency data were collected.

## Results

### The Performance of the Super-Resolution Imaging With Simulations

**Figure 4:**
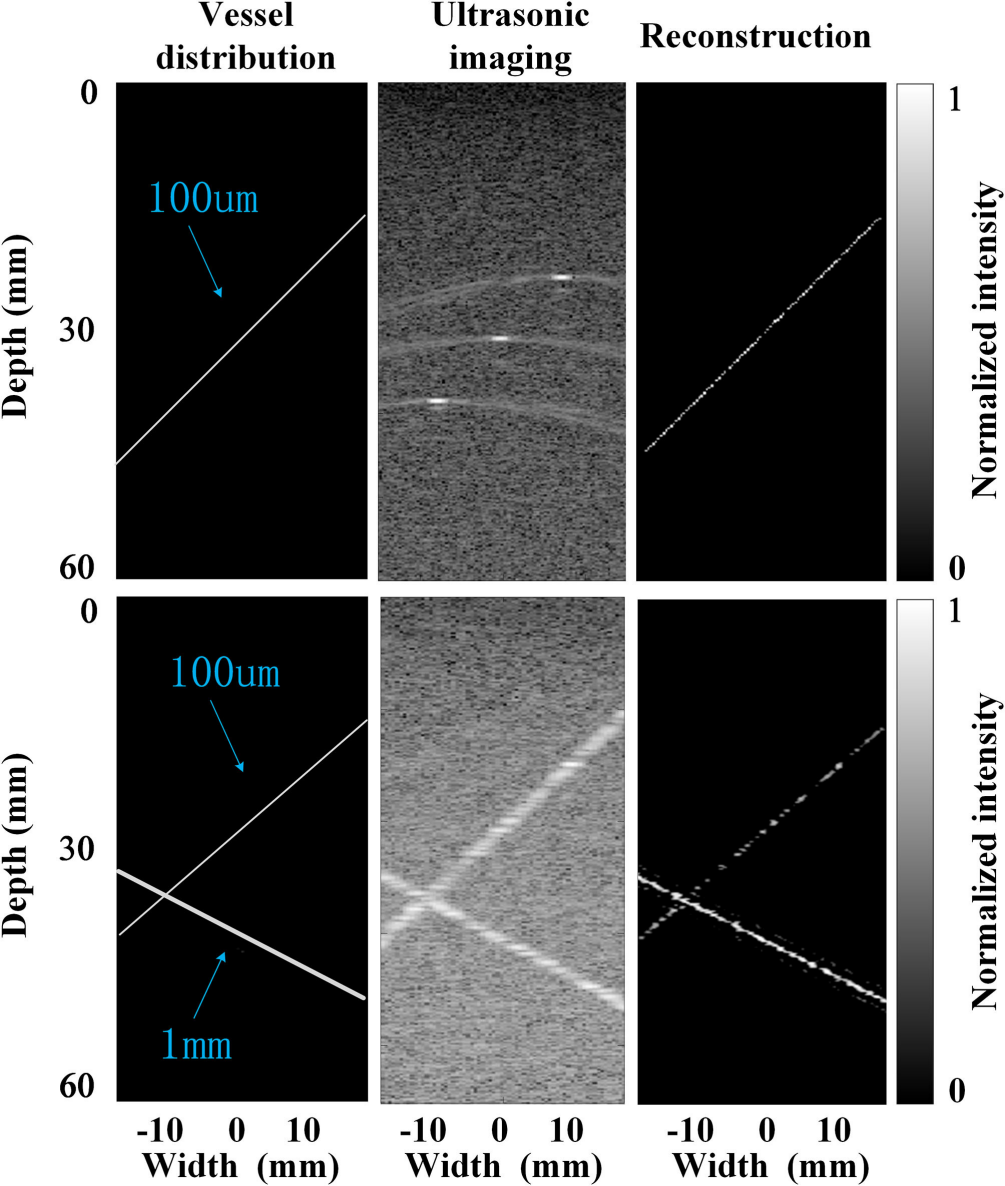
Simulations with very low bubble density (up row, an initial concentration of 1 × 10^2^ MBs per ml) and high bubble density (bottom row, an initial concentration of 2 × 10^9^ MB per ml), respectively.

The accuracy of this super-resolution imaging method was firstly evaluated by simulation. Specifically, an ultrafast compounded plane waves (CPW) imaging sequence with five tilted chirp waves (CCW-5, −6°, −3°, 0°, 3°, and 6°, PRF = 1,500 Hz) was transmitted in the forward direction along with the entire aperture by Field II simulation (18), and radio-frequency (RF) echoes were received at 128 channels from a modeled phantom placed in an area of 0 mm width and 60 mm depth, containing vessels with different diameters, which can be seen in Figure 4. Once 600 frames of the RF data were acquired, the previously used spatiotemporal filtering technique was implemented on top of the ultrafast imaging stack to discriminate the high temporal components of the flowing MBs. Then, the MCMCDA tracking algorithm was applied to acquire the super-resolution imaging result. For the quantitative estimation of the reconstructive errors, the common image quality evaluation indexes, the mean squared error (MSE) (19) was chosen as the error measurement. Specifically, the MSEs comparing the designed vessel distributions and the proposed method reach 0.08 and 0.09 for the low and high bubble densities, respectively (the smaller the value of the MSE indexes, the smaller the distortion of the reconstruction), which illustrates the accuracy of the two methods.

### Further Evaluation by Phantom Experiments

**Figure 5:**
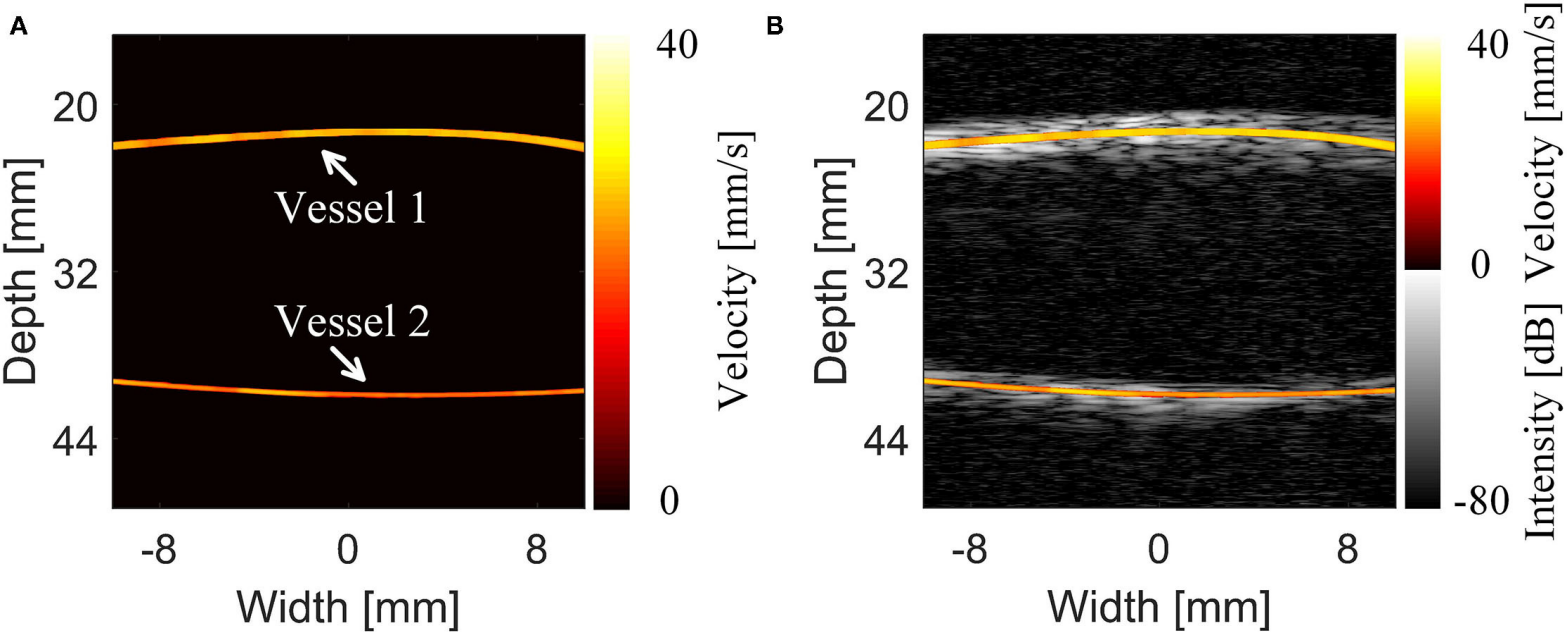
Results and comparison of the super-resolution imaging results of the cerebrovascular phantom (14). **(A)** Super-resolution imaging results of the phantom. **(B)** The compound image of the super-resolution imaging results and 5-angle compound chirp waves (CCW-5) results.

The MB tracking results and the coordinate-based flow rate information are provided in Figure 5. The flow rate was acquired by superposing and averaging the velocities of the MBs at each pixel within the continuous flow time. The imaging result displayed the morphology and structure of the vessel and the mean blood flow velocity along the vessel. Ackermann et al. (8) demonstrated that with this imaging method, the respective trajectories of the different MBs passing through the same vascular segment can be regarded as a set of parallel lines. Given that thicker vessels are associated with higher blood flow velocities, Figure 5A shows that the mean blood flow velocity in the large vessels (inner diameter = 1 mm) is slightly greater than that in the microvessels (inner diameter = 0.7 mm). Subsequently, Figure 5B shows a compound image superimposing the estimated motion trajectories of the MBs and the raw contrast-enhanced imaging results. This trajectory result exactly matches the contrast-enhanced imaging result and sufficiently describes the detailed information of the vascular structure. Therefore, this method can achieve high-precision and high-accuracy imaging results in *in vivo* animal experiments.

### Imaging Results With Rhesus Monkeys

The super-resolution imaging method proposed by the research group at the Xi'an Jiaotong University was used to reconstruct the MB flow trajectory in the blood on a grid with 10 × 10 μm cells to realize the super-resolution imaging of the intracranial capillary network in rhesus monkeys.

**Figure 6:**
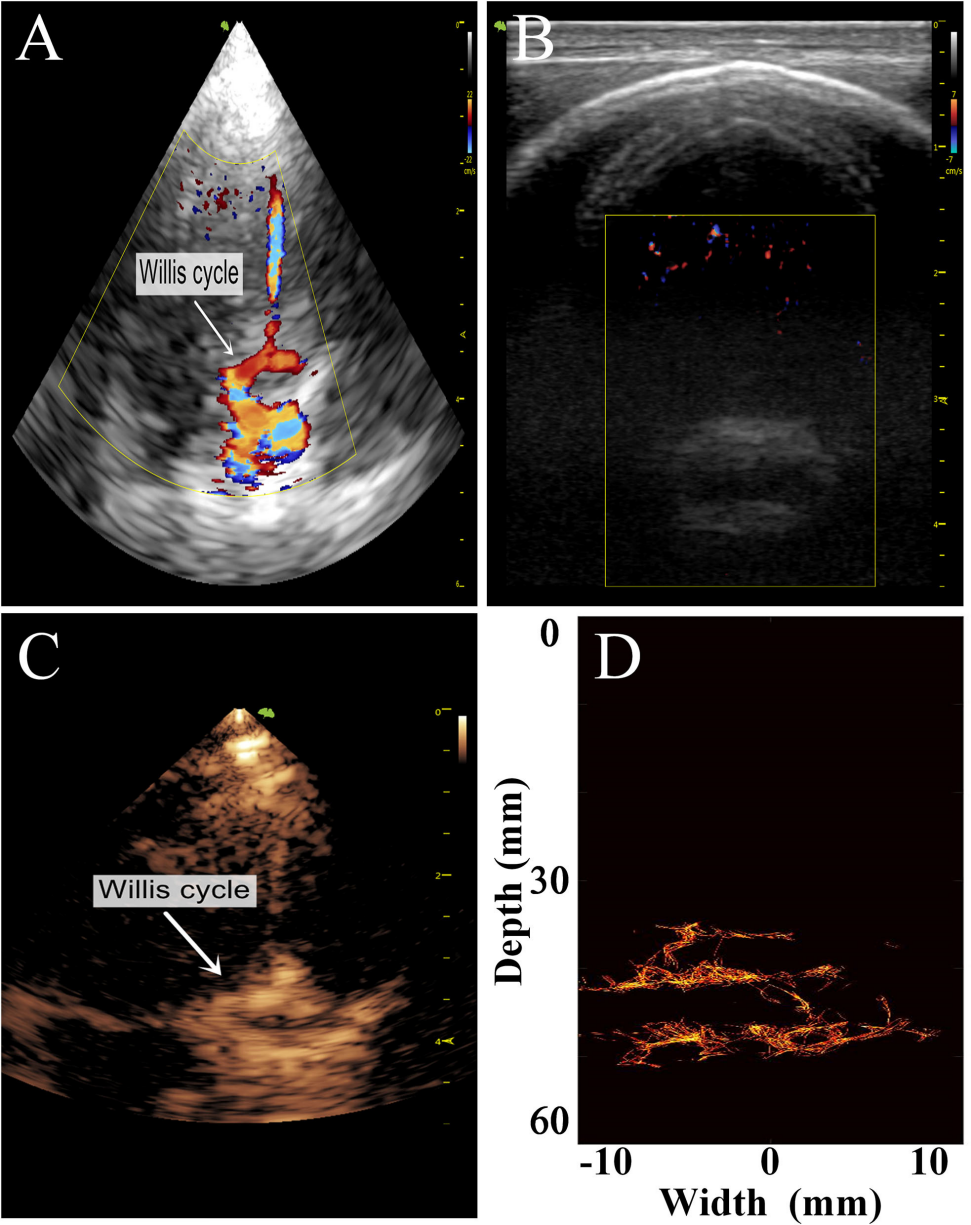
Comparison of images of the transparietal region in rhesus monkeys obtained by different imaging methods. **(A)** Low-frequency phased-array imaging. **(B)** High-frequency linear-array imaging. **(C)** Conventional contrast-enhanced ultrasound. **(D)** Super-resolution imaging results.

**Figure 7:**
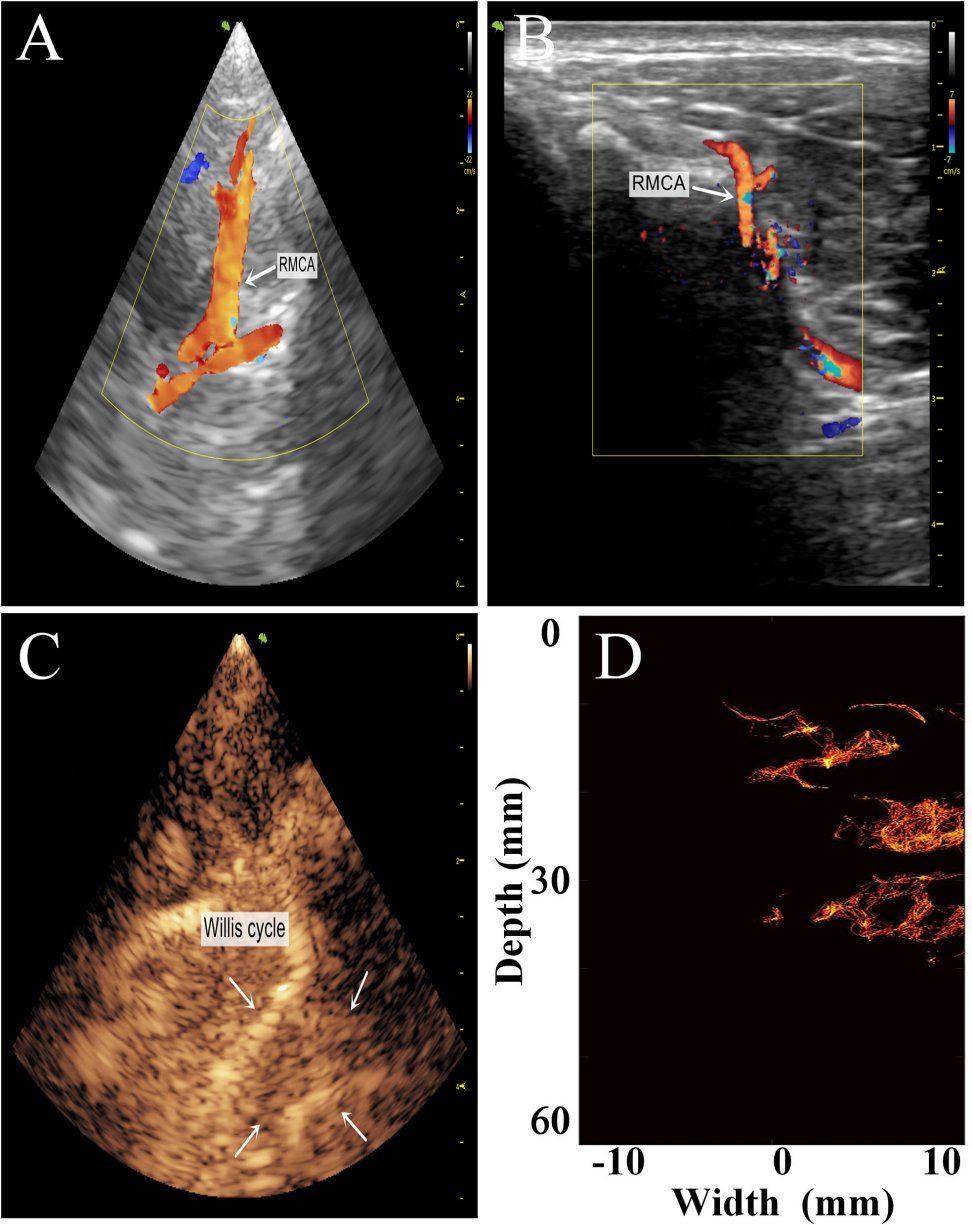
Comparison of the transtemporal window images of the rhesus monkeys with different imaging methods. **(A)** Low-frequency phased-array imaging. **(B)** High-frequency linear-array imaging. **(C)** Conventional contrast-enhanced ultrasound. **(D)** Super-resolution imaging results. RMCA, right middle cerebral artery.

To preliminarily evaluate the imaging effectiveness, we selectively scanned the characteristic intracranial structures, including the cerebral arterial circle, the anterior, middle, and posterior cerebral arteries, which were subjected to super-resolution imaging after target localization by CEUS (Figures 6, 7).

The imaging results are not satisfactory because most vascular details were lost due to skull shielding. The CEUS shows only the areas with extensive MB activity in the monkey brain, which are enhanced and imaged.

**Figure 8:**
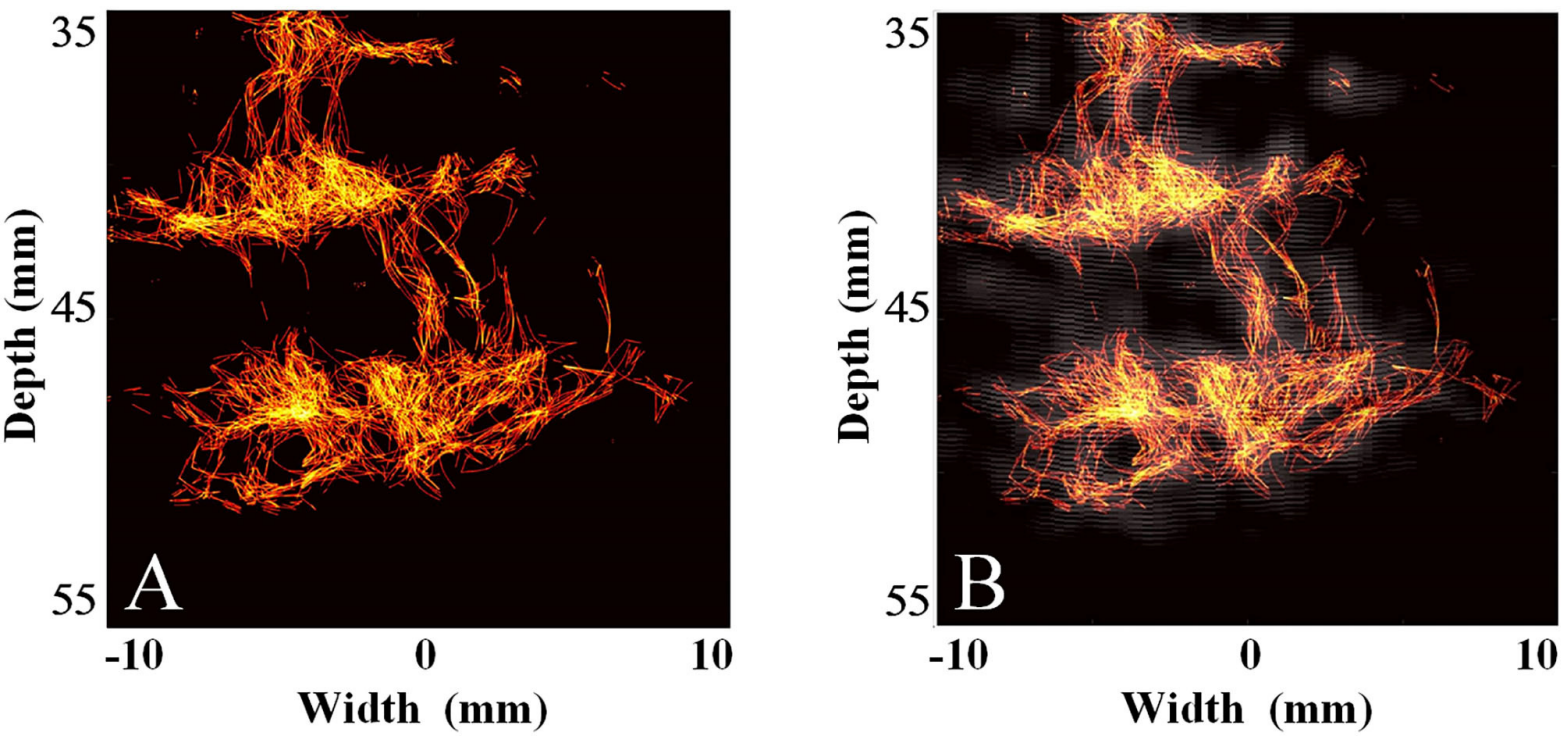
Super-resolution transparietal imaging results of the cerebral arterial circle in rhesus monkeys. **(A)** Super-resolution imaging results. **(B)** The compound image superimposing super-resolution imaging results and STF imaging results.

**Figure 9:**
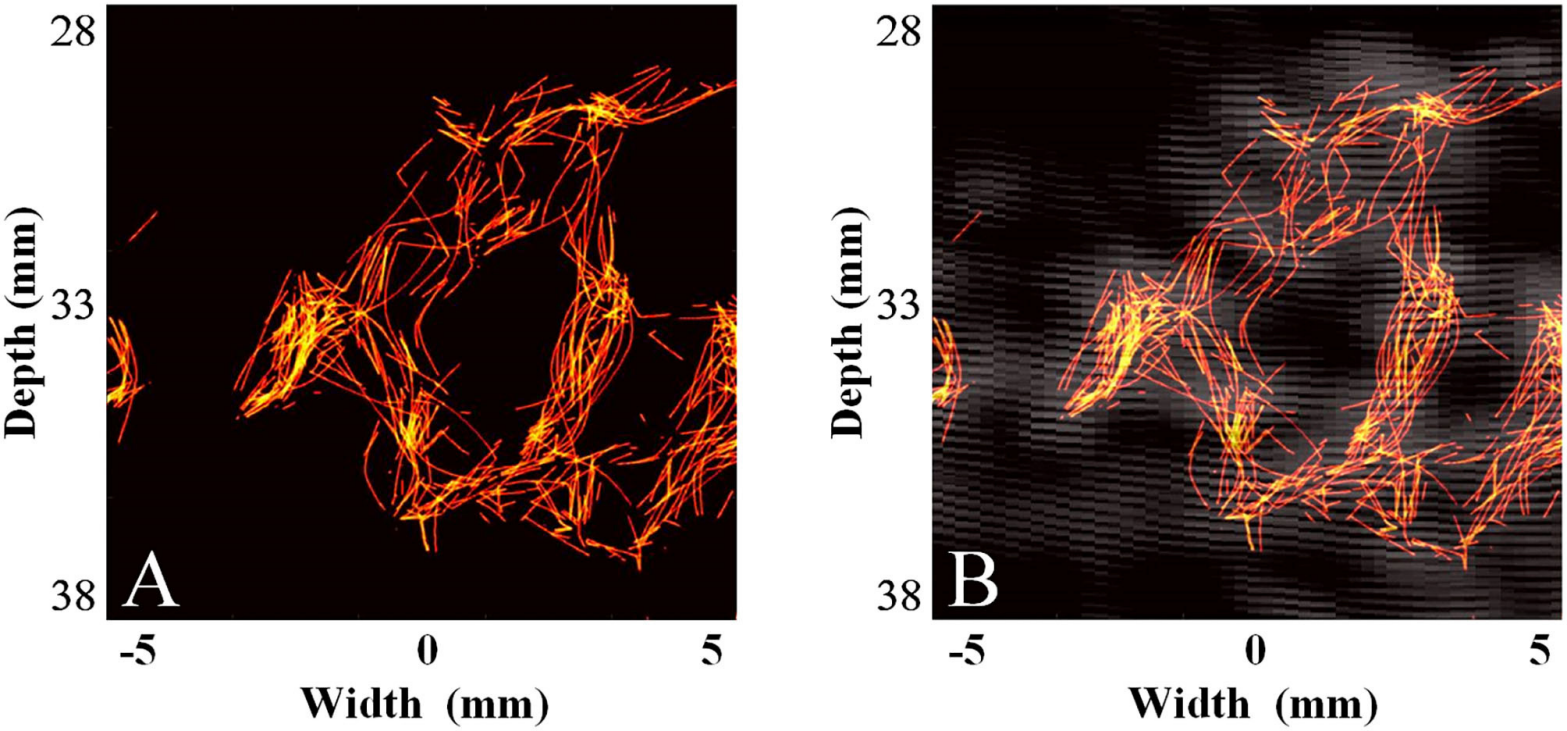
Super-resolution transtemporal imaging results of the cerebral arterial circle in rhesus monkeys. **(A)** Super-resolution imaging results. **(B)** The compound image superimposing super-resolution imaging results and STF imaging results.

Subsequently, we used the MCMCDA high-concentration MB tracking method proposed by the research group at the Xi'an Jiaotong University, to reconstruct the intracranial microvessels in the rhesus monkeys and obtained extensive details for the intracranial capillary distribution. Compared with the conventional low-frequency ultrasound imaging, the super-resolution reconstruction of the microvascular structures was completed using a minimum pixel size of 10 × 10 μm (Figures 8, 9).

## Discussion

Due to the severe attenuation of the ultrasound signal caused by skull shielding, the detection rate of MBs is substantially reduced, and the hypointense signals are presented. Although the low-frequency linear frequency-modulated signals can penetrate the skull and yield ultrasonic images, the image quality is still far from the requirements for displaying intracranial microvessels, even if the strong harmonic components of the MB signals are extracted through a spatial-temporal filter (STF) when affected by the low imaging resolution of the low-frequency probe and the enhancement of echo signals by linear frequency-modulated signals. In this condition, the super-resolution imaging method proposed by the research group at the Xi'an Jiaotong University was used to reconstruct the MB flow trajectory in the blood.

Compared with super-resolution optical fluctuation imaging (SOFI) (9), which cannot provide blood flow velocity information, the proposed MCMCDA high-concentration MB tracking algorithm can yield more accurate velocity information. In addition, compared with those required for ultra-fast ultrasonic MB tracking microscopy imaging, the data acquisition volume and acquisition time required by our method are greatly reduced, thus, the errors caused by respiration and other movements can be better avoided.

The large inner diameter and fast blood flow velocity (up to ~100 cm/s) of the large intracranial vessels in rhesus monkeys far exceed the upper limits of the tracking ability of the super-resolution imaging algorithms. In addition, because the algorithm is tailored for the imaging of microvessels <1 mm in size, it can only acquire information for slow blood flows in the large vessels, resulting in low vessel filling and cluttered blood flow trajectories.

As shown by the comparison between the images of the intracranial vessels in rhesus monkeys obtained using different imaging methods, the super-resolution imaging technique can reveal blood flow information in the microvessels that cannot be captured by conventional ultrasound imaging methods, providing more abundant microvessel information. However, this technique has a weak ability to capture blood flow motion in large vessels and cannot adequately distinguish the vascular boundaries or accurately describe the flow velocity information. Therefore, the method can only be used for qualitative determination rather than the quantitative analysis of blood flow.

The super-resolution microvascular ultrasound imaging technique is rapidly emerging and developing. The unprecedented combination of high imaging resolution and permeability has facilitated extensive preclinical and clinical application research (20, 21). Conventional ultrasound imaging techniques are limited in terms of both spatial and temporal resolution (1, 22, 23). Current studies have shown that the MB localization-based super-resolution imaging technique has demonstrated a potential for maximizing the spatial resolution beyond acoustic diffusion limits (9, 10, 24).

Contrast-enhanced super-resolution ultrasound imaging, also known as ultrasound localization microscopy, can identify vessels smaller than the diffraction limit and has recently shown the ability to display super-resolution vascular images of shallow body structures in small animals. To fully translate this technique into clinical application, MBs should be detected at deeper locations in tissues while maintaining a short acquisition time. Currently, this imaging method relies on plane-wave imaging, with the advantage of the maximum frame rate, which is important for the requirement of a large number of frames by super-resolution processing. However, the method also causes a poor contrast ratio of the wide-plane beam for field irradiation and has low sensitivity for bubble detection.

This method is non-invasive and highly sensitive for MB detection compared with other brain microvessel imaging methods, such as ultra-fast ultrasonic MB tracking microscopy imaging and SOFI. Moreover, with this method, images of brain microvessels with micron-scale resolution and flow velocity information are available based on a reasonable data acquisition time. Overall, super-resolution imaging can non-invasively produce intracranial microvessel images with micron-scale resolution and accurate blood flow velocity information. Because of its high potential application value, the method can be clinically applied for the auxiliary diagnosis of craniocerebral diseases with abnormal blood flows in microvessels, such as cancer and stroke (25). This imaging method with high spatiotemporal resolution can provide a basis for subsequent MB-based imaging research and serve as an important reference for clinical applications of non-invasive transcranial ultrasound.

In terms of the limitation, the main factor affecting the detection time for the number of positions and the execution time in the MCMCDA algorithm is the image size. Although the current implementation was not suitable for real-time processing, in our future work we intend to improve the execution results. As radar tracking algorithms with similar complexities have been widely used in different real-time applications, real-time tracking of multiple MBs for microvessel reconstruction and blood flow reconstruction in cerebral vessel imaging is promising. Moreover, in the future, graphic processing unit (GPU) acceleration has the potential to decrease the time consumption of the method to implement real-time cerebral vessel imaging with satisfactory spatiotemporal resolution.

As a frontier imaging method still in the exploratory stage, super-resolution ultrasound imaging shows the potential to visualize microvascular details at the capillary level (i.e., subwavelength resolution) but still requires optimization (26). Little experimental evidence is available for the application of super-resolution ultrasound imaging of brain vessels in large animals, and inevitably, some aspects of its application methods and result processing must be improved, such as the localization and real-time problems, as well as artifacts and error estimation. At present, most research on the novel technique of super-resolution imaging focuses on the theoretical level, and the relevant studies are mostly in the field of engineering, with few in the field of medicine. The imaging data obtained in this study were mainly derived or deduced based on theoretical knowledge, which lacks the support of clinical data as controls. In the near future, super-resolution imaging is expected to demonstrate its advantages in the ultrasound community and to better serve clinical needs with the continuous dissemination, application, and popularization of this novel technique in the medical field.

## Data Availability Statement

The original contributions presented in the study are included in the article/supplementary material, further inquiries can be directed to the corresponding author/s.

## Ethics Statement

The animal study was reviewed and approved by the Institutional Animal Care and Use Committee of the Air Force Medical University.

## Author Contributions

LY and CB conceived and designed the study. LY wrote the paper. MW and YZon acquired the datas. CB and XZ analyzed and interpreted datas. YZhe reviewed and edited the manuscript. SC and YZho revised the manuscript. All authors contributed to the article and approved the submitted version.

## Funding

This research was supported by the Fundamental Research Funds for the Central Universities (Grant No. G2021KY05107) and the Youth Innovation Promotion Association, CAS (Grant No. 2021401).

## Conflict of Interest

The authors declare that the research was conducted in the absence of any commercial or financial relationships that could be construed as a potential conflict of interest.

## Publisher's Note

All claims expressed in this article are solely those of the authors and do not necessarily represent those of their affiliated organizations, or those of the publisher, the editors and the reviewers. Any product that may be evaluated in this article, or claim that may be made by its manufacturer, is not guaranteed or endorsed by the publisher.
